# Targeting epigenetic alterations in cancer stem cells

**DOI:** 10.3389/fmmed.2022.1011882

**Published:** 2022-09-20

**Authors:** Francesco Verona, Vincenzo Davide Pantina, Chiara Modica, Melania Lo Iacono, Caterina D’Accardo, Gaetana Porcelli, Dario Cricchio, Alice Turdo, Miriam Gaggianesi, Simone Di Franco, Matilde Todaro, Veronica Veschi, Giorgio Stassi

**Affiliations:** ^1^ Department of Health Promotion, Mother and Child Care, Internal Medicine and Medical Specialties (PROMISE), University of Palermo, Palermo, Italy; ^2^ Department of Surgical, Oncological and Stomatological Sciences (DICHIRONS), University of Palermo, Palermo, Italy

**Keywords:** cancer stem cells, epigenetic alterations, epigenetic inhibitors, regenerative medicine, normal stem cells

## Abstract

Oncogenes or tumor suppressor genes are rarely mutated in several pediatric tumors and some early stage adult cancers. This suggests that an aberrant epigenetic reprogramming may crucially affect the tumorigenesis of these tumors. Compelling evidence support the hypothesis that cancer stem cells (CSCs), a cell subpopulation within the tumor bulk characterized by self-renewal capacity, metastatic potential and chemo-resistance, may derive from normal stem cells (NSCs) upon an epigenetic deregulation. Thus, a better understanding of the specific epigenetic alterations driving the transformation from NSCs into CSCs may help to identify efficacious treatments to target this aggressive subpopulation. Moreover, deepening the knowledge about these alterations may represent the framework to design novel therapeutic approaches also in the field of regenerative medicine in which bioengineering of NSCs has been evaluated. Here, we provide a broad overview about: 1) the role of aberrant epigenetic modifications contributing to CSC initiation, formation and maintenance, 2) the epigenetic inhibitors in clinical trial able to specifically target the CSC subpopulation, and 3) epigenetic drugs and stem cells used in regenerative medicine for cancer and diseases.

## Epigenetics and normal vs. cancer stem cells

### What’s epigenetics and how important is it in normal and cancer stem cell biology?

Although in the eukaryotic organism all cells contain the same DNA sequence, different cell types in distinct tissues perform different functions. This potential is determined by the regulation of gene expression. Epigenetics is a regulatory mechanism of gene expression that does not lead to alterations of DNA sequence but operates on the physical structure of DNA or histones, the DNA associated proteins. In eukaryotes, octameric histones compact DNA in a higher order and dynamic 3D structure, called chromatin, which is acquired in a progressive manner through covalent modifications and is therefore reversible. At the basal level chromatin is formed by nucleosomes, where two turns of DNA (146 base pairs) are wrapped on histone octamers formed by two copies of each core histone H3, H4, H2A and H2B. Histone H1 binds nucleosomes to each other, further compacting the DNA molecule into a 30 nm fiber. The dynamism of epigenetic regulation allows cells to adapt and respond to external stimuli and has a central role in different cellular processes, including transcription, DNA repair, replication and cellular differentiation. The epigenetic information not contained in the DNA sequences is heritable and pass on from mother to daughter cells. Alterations in epigenetic regulation are hallmarks of senescence and many diseases, including fragile X syndrome and cancer (Okano et al., 1999; Bond et al., 2015; Gupta et al., 2019). Of note, in cancer despite the advanced knowledge and continuous research on driver mutations, recent studies on the non-genetic determinants confirmed that these epigenetic mechanisms are involved in chromosomal instability, oncogene activation, silencing of oncosuppressor genes and also in the development of tumor heterogeneity. Interestingly, several aberrant epigenetic alterations have been associated and linked to the determination and formation of a subpopulation of cells known as cancer stem cells (CSCs), promoting tumor initiation (Vicente-Duenas et al., 2018; Kumar et al., 2022). Indeed, tumor tissues seem organized hierarchically, at the top of which there are CSCs (Sato et al., 2003; Kreso and Dick, 2014). This population has been identified in many tumor types such as breast, colon, thyroid, brain, ovary, prostate, pancreas, liver, skin and lung cancers, and show many features, tumor-initiating capability, self-renewal, DNA repair, high density of drug transporter and thus therapy resistant mechanisms (Todaro et al., 2007; Turdo et al., 2019; Turdo et al., 2020). In addition, CSCs are considered responsible for the metastatic process (Medema, 2013; Todaro et al., 2014; Veschi et al., 2020; Gaggianesi et al., 2021). Each epigenetic mechanism has its purpose in lesser or greater compactness of the chromatin, determining accessibility (euchromatin) to the RNA polymerase and to all transcriptional factors. In particular, epigenetic mechanisms include events that modify the elements of chromatin, DNA and histone proteins. DNA methylation is the best-known epigenetic process, which regulates chromatin remodeling and gene expression, thus determining several biological processes such as differentiation and genomic stability (Atlasi and Stunnenberg, 2017; Park et al., 2022). Differentiated cells showed a stable and unique DNA methylation pattern (Moore et al., 2013). The addition and maintenance of methyl groups on cytosine residues to form 5-methylcytosine (5mC) in CpG dinucleotides, called CpG sites, are orchestrated by a family of DNA methyltransferases (DNMTs). In particular, DNMT3A and DNMT3B are responsible for the *de novo* methylation of DNA, a heightened process in embryonic cells and development (Okano et al., 1999). It has been shown that altered methylation due to the lack of these enzymes is also associated with an alteration of the *OCT4* and *NANOG* stem genes (Li et al., 2007; Bibikova et al., 2008). The characterization of ten-eleven translocation protein 1 (TET1) and its role shed new lights on the complexity of DNA methylation and in particular how it could be erased (Tahiliani et al., 2009), although until recently it was considered an irreversible process (Rasmussen and Helin, 2016). Additionally two enzymes from the same family, TET2 and TET3, were identified. All these three enzymes catalyze the oxidation of 5 mC, thus inducing the formation of 5-hydroxymethylcytosine (5hmC), 5-formylcytosine (5fC) and 5-carboxylcytosine (5caC) (Jiang, 2020). It has been demonstrated that alterations on gene expression profiles of these enzymes appear to be involved in many cancer types (Pan et al., 2015; Xu et al., 2019).

Histone modifications include phosphorylation, acetylation (Ac), crotonylation, methylation (Me), sumoylation, and mono-ubiquitination (Ub) on the selected amino-terminal tail, lysines (K), arginines (R), and serines (S) that are catalyzed by proteins engaged by transcription machinery (Berger, 2007). Histone changes occur following a precise order, which is referred to as the “histone code” as it regulates the chromatin condensation and the accessibility of transcription systems (Strahl and Allis, 2000). In particular, histone methyltransferases (HMTs) and histone acetyltransferases (HATs) add methyl or acetyl groups to the histone tails, respectively, whereas histone demethylases (HDMs) and histone deacetylases (HDACs) remove these groups. Modifications affecting histone H3 and its biological significance have been extensively studied in CSCs compared to histone H2 and histone H4 (Chi et al., 2010). In particular, the post-transcriptional modifications of histone H3 such as H3K27me3, H3K27ac and H3K4me3, are responsible for plastic and dynamic chromatin maintenance in CSCs. To support these data, it was demonstrated that during the cellular differentiation, stem cells (SCs) lose H3K4me3 mark and acquire a compact chromatin profile (Suva et al., 2013; Yamazaki et al., 2013). While acetylation is usually linked to a chromatin opening and gene activation, the role of histone methylation depends on the histone in which it occurs, in fact, both events could lead to the induction or repression of gene transcription. For example, mono-methylation of lysine 4 on histone H3 (H3K4me1) in the promoter region is associated with a limited recruitment of specific enzymes involved in chromatin remodeling (Cheng et al., 2014), while di- and tri-methylation (H3K4me2, H3K4me3) are generally linked to a transcriptionally active form of chromatin (Pekowska et al., 2011). Trimethylation of lysine 9 or 27 on histone H3 (H3K9me3 or H3K27me3), are associated with regions of inactive gene transcription (Barski et al., 2007; Eissenberg and Shilatifard, 2010). Of note, many histone modifying enzymes display non histone targets which may play a crucial role in modulating the epigenetic reprogramming of pediatric cancers derived from neuroblasts, supporting their potential role in affecting also the plasticity of the stem cell compartment (Veschi et al., 2017; Veschi and Thiele, 2017; Veschi et al., 2019).

Another important change affecting histones is the incorporation of histone variants. The histone variants can totally replace the canonical histones or form heterotypic nucleosomes with them. Histone genes can be classified as replication dependent histones (S phase), which are known as canonical histones, tissue specific histones and cell cycle replication independent histones. The latter two types are known as histone variants, which differ from canonical in the moment of deposition on chromatin and the amino acid sequence. For example, human embryonic stem cells (hESCs) exhibit higher levels of H1.1, H1.3, and H1.5 compared with specialized cells while the variant H1.0 is involved in cell differentiation (Terme et al., 2011). One of the most common variant of H2A histone is macroH2A, which is correlate with a repression of gene transcription, that replaces the canonical H2A in inactivated X chromosomes of female cells and senescence process (Zhang et al., 2005). Furthermore, macroH2A is upregulated during differentiation processes of embryonic and adult SCs (Barrero et al., 2013).

In 2012, H2A.Z-H2A hybrid couple has been shown as highly expressed in mouse SCs in particular localized at the transcriptional start sites (TSS) of expressed genes (Nekrasov et al., 2012). In addition, the variant H2A.Z has been studied in cancer progression and it has been associated to increased risk of metastasis in ERα-positive breast cancer patients (Hua et al., 2008). Compelling evidence highlighted the fundamental role of H2A.Z in ESC development and in regulating the gene expression patterns of specialized cells (Subramanian et al., 2013).

Non-coding RNA (ncRNA) are RNA sequences that are transcribed but not translated into proteins. They can be classified according to their length in small RNA and long ncRNA, of about 20–22 or 200 nucleotides, respectively. NcRNAs have recently been reported to interact with nucleic acids and proteins. Small ncRNAs usually result in downregulation/silencing of gene expression, while long ncRNAs (lncRNAs) may have structural and regulatory functions, and they can act both in nucleus and in cytosol, interacting with DNA, RNA or proteins, leading to different effects on the regulation of biological processes (Statello et al., 2021). Interestingly, lncRNAs are cell-type specific and play important roles in SC maintenance and differentiation (Statello et al., 2021).

All this evidence points out an urgent need to better understand the epigenetic mechanisms, that influence the transition of normal versus cancer SCs and their characteristic features including their tumorigenic capacity and metastatic potential.

### Normal versus cancer stem cells

The above-described mechanisms of epigenetic regulation are essential and crucial for the future of SCs. Normal stem cells (NSCs) are responsible for different biological processes such as embryonic development and tissue homeostasis (Rossi et al., 2020), and they are characterized by limited self-renewal. These cells can undergo a particular cell division giving rise to two SCs with the same characteristics, to maintain the SC pool, and another division that leads to one SC and one progenitor cell to continue the differentiation process (Fuchs and Chen, 2013). Regenerating and repairing organs, as well as maintaining normal tissue homeostasis and differentiating into specialized cells under specific signals, represent additional functions of NSCs. NSCs display a diploid genome, are generally quiescent and characterized by a restricted proliferation potential (Voog and Jones, 2010). Tissue SCs create continuous crosstalk with their microenvironment, called niche. The released niche factors regulate, at the epigenetic level, the fate of SCs (Wu and Sun, 2006). SCs are classified based on their differentiation potential, into *totipotent SCs* that are able to generate all cell types, including embryonic and extraembryonic tissue (for example morula), *pluripotent SCs* generate all body cells excluding extraembryonic tissue (such as blastocyst), *multipotent SCs* that have the ability to develop specific cell types (such as hematopoietic cells), and *unipotent SCs* that have an aptitude to generate exclusively single cell types (for example hepatic cells) (Mardanpour et al., 2008). The stemness potential and the differentiation status of progenitor cells are determined by the epigenetic changes, which include DNA methylation and histone modifications. The pluripotent cells have a different chromatin configuration from the other cells they present the chromatin lightly packed and permissive for an active gene transcription. During the differentiation process, stem genes turn off, while only specialized genes of committed cells became transcriptionally active (Stergachis et al., 2013; Atlasi and Stunnenberg, 2017).

A study regarding the genome-wide distribution of DNA methylation highlighted that the methylation of CpG islands undergoes modifications during cellular differentiation. This event drives SCs towards their differentiation fate (Meissner et al., 2008). Generally, CpG islands remain unmethylated in NSCs, however, there are differentially methylated regions (DMRs) such as those present in the inactivated X chromosome. The differentiation process leads to an increase in methylated areas with a reduction of the multipotent capacity and self-renewal through gene silencing (Bibikova et al., 2008; Fuchs and Blau, 2020). It is known that the expression pattern of pluripotency is regulated by the transcription factors Oct4, Nanog and Sox2, when these factors are reintroduced into specialized cells these cells can reprogram themselves (Li et al., 2012). Accordingly, it has recently been shown that TET1 and TET2, enzymes that demethylate DNA, are expressed in mouse ESCs (Ito et al., 2010) and are Oct4-regulated, supporting the pluripotent state (Koh et al., 2011). Since the discovery of stem cell-like cells in patients affected by leukemia, a novel theory of tumor initiation was postulated regarding the presence within the tumor of CSCs derived from NSCs, that have underwent abnormal epigenetic alterations. Therefore, upon the epigenetic reprogramming CSC subpopulation, defined by uncontrolled proliferation and self-renewal capacity, has been considered responsible for tumor initiation, progression and therapy resistance (Turdo et al., 2019). In summary, in the majority of cases scientists believe that CSCs, derive from NSCs that have lost the control of proliferation processes, present deregulation of common Wnt/β-catenin, JAK-STAT, TGF-β and hedgehog (HH) signaling pathways, or have undergone mutation and/or abnormal epigenetic alteration (Rycaj and Tang, 2015).

An important characteristic to distinguish NSCs versus CSCs is represented by the chromatin accessibility. Nowadays, despite the gold standard methods used in the past decades, multiple complex biochemical techniques have been developed to study chromatin accessibility and to design maps of the chromatin status across a large number of tissues and cell types in several diseases, including cancer. Specifically, high-throughput technology, through the evaluation of cis-regulatory sequences analysis, the adoption of single-cell methods together with in silico and bioinformatic approaches allow the exploration of chromatin accessibility profiles both in bulk population and at a single-cell level. Here, we will discuss the innovative high-throughput technologies applied for the study of chromatin accessibility in NSCs and CSCs. RNA expression with sequencing (SHARE-seq) is used for single or several measures of chromatin accessibility at a single-cell level and the relative gene expression profile. Of note, this technology allows the study of the trajectory lineage for each cell. Ma et al. (2020), studied the chromatin potential state of a single cell to its future RNA states, identifying the cell fate which may follow during developmental transitions by SHARE-seq analysis application. Additionally, transposase-accessible chromatin with high-throughput sequencing (ATAC-seq) has been used to investigate chromatin states transition in early human pre-implantation development. Particularly, ATAC-seq, through integrative analysis allows studying the conservation or divergence in regulatory circuitry during early stage of embryonic development both in human and mouse and, between human pluripotent stem cells *in vivo* and hESCs (Wu et al., 2018). Single-cell chromatin accessibility methods allow also the identification of a stem-like cell subpopulation in primary tumors starting from the bulk population. Specifically, by the application of ATAC-seq technique, Guilhamon et al. demonstrated that the transcription factor SP1 promotes stemness and invasion in glioblastoma and that FOXD1, a pluripotency transcription factor, controls the expression of the aldehyde dehydrogenase ALDH1A3, a marker of invasive potential in glioblastoma SCs (Guilhamon et al., 2021). Finally, formaldehyde-assisted isolation of regulatory elements sequencing (FAIRE-seq) is an emerging technology applied to identify regulatory elements distant from their gene targets. FAIRE-seq consists of a silico approach to predict transcription factors binding sites and a high-throughput sequencing of a plethora of cell types, aimed to evaluate the complexity of several transcriptional networks focusing on biological processes such as epithelial–mesenchymal transition (EMT) and CSC formation. The FAIRE-seq application in breast cancer models highlighted regions with increased accessibility. Specifically, FOX and AP-1 regions are associated with increased expression of CSC-associated genes. On the other hand, the transcriptional repression of two FOX family members, FOXN2 and FOXQ1, impairs CSC formation and reduces the expression of stemness-related genes (Hardy et al., 2016). Elucidating the differences in chromatin accessibility between NSCs and CSCs by the above-mentioned high-throughput techniques will help to identify novel potential biomarkers/therapeutic targets specific for this aggressive subpopulation.

In the following paragraphs we will review the principal epigenetic mechanisms and/or alterations related to the initiation and maintenance of CSCs.

## Epigenetic alterations critical for cancer stem cell initiation

Despite the effort employed in the study of cancer biology, the specific trigger that induces cell transformation and tumor initiation is still unclear (Vicente-Duenas et al., 2013). In the past, the study of biological processes related to the biology of cancer has been focused on finding molecular pathways and genetic alterations, at the SC compartment level, trying to shed light on the molecular basis responsible for initiation, promotion and progression of cancer. In the recent years there has been an increasing emphasis on the characterization of the epigenetic mechanisms associated with cancer initiation and evolution (Kumar et al., 2022). Many works have shown that epigenetic alteration are implicated in numerous steps during cancer initiation, being responsible for abnormal expression of oncogenes and tumor suppressor genes, thus leading to tumor transformation (Cheng et al., 2019). In particular, SCs and progenitors are characterized by specific epigenetic profiles, which make them more susceptible to acquire DNA mutations than differentiated cells (Beerman and Rossi, 2015). Both cell-intrinsic (i.e., mutations) and cell-extrinsic (i.e., environmental cues) factors control the epigenetic status of cancer cells. Specifically, epigenetic alterations occurring in the SC compartment may be amplified within the cell compartment, bringing a selective advantage on cell growth and maintenance. During this process, further epigenetic changes or additional alterations at the genetic level that lead to cellular transformation may also occur. These changes are inherited from the daughter cells, resulting in a pool of cells that support tumor initiation (Beerman and Rossi, 2015). Gene expression is dramatically altered in CSCs compared to healthy cells resulting from an aberrant modulation of the epigenetic machinery. Hypermethylation of CpG islands and silencing of tumor suppressor genes and/or pro-differentiation factors are characteristic epigenetic traits of CSCs. Of note, the epigenetic status of malignant ESCs is significantly different regarding the methylation profile compared with CSCs in adult cancers (Ohm et al., 2007).

Among several theories proposed in order to explain the origin of CSC, further hypotheses have been postulated about alternative mechanisms contributing to CSC formation or other precursor cells from which CSC may derive, beyond NSCs. According to the “cell reversal theory” (CRT), a somatic cell can dedifferentiate after a specific perturbation (a potential carcinogenic event), in this way the cell switches to a different epigenetic state which could activate an uncontrolled proliferation. A cell can enter on the pathological\cancerous epigenetic program and lead to the formation of what is labeled as a CSC (Carvalho, 2020). Similarly to CRT, Friedmann-Morvinski and Verma presented a theory about the origin of CSC resulting from tumor progression. Specifically, they explained that upon reprogramming into a pluripotent state that could drive tumor progression, the dedifferentiation of tumor cells could determine CSC formation *via* epigenetic resetting (Friedmann-Morvinski and Verma, 2014). Moreover, Nimmakayalaa et al. (2019) hypothesized that CSC origin could be driven by cell fusion, horizontal gene transfer, exposure to hypoxia and toxic agents, metabolic reprogramming or mutations in differentiated cells that guide the reprogramming of them into CSC.

**FIGURE 1:**
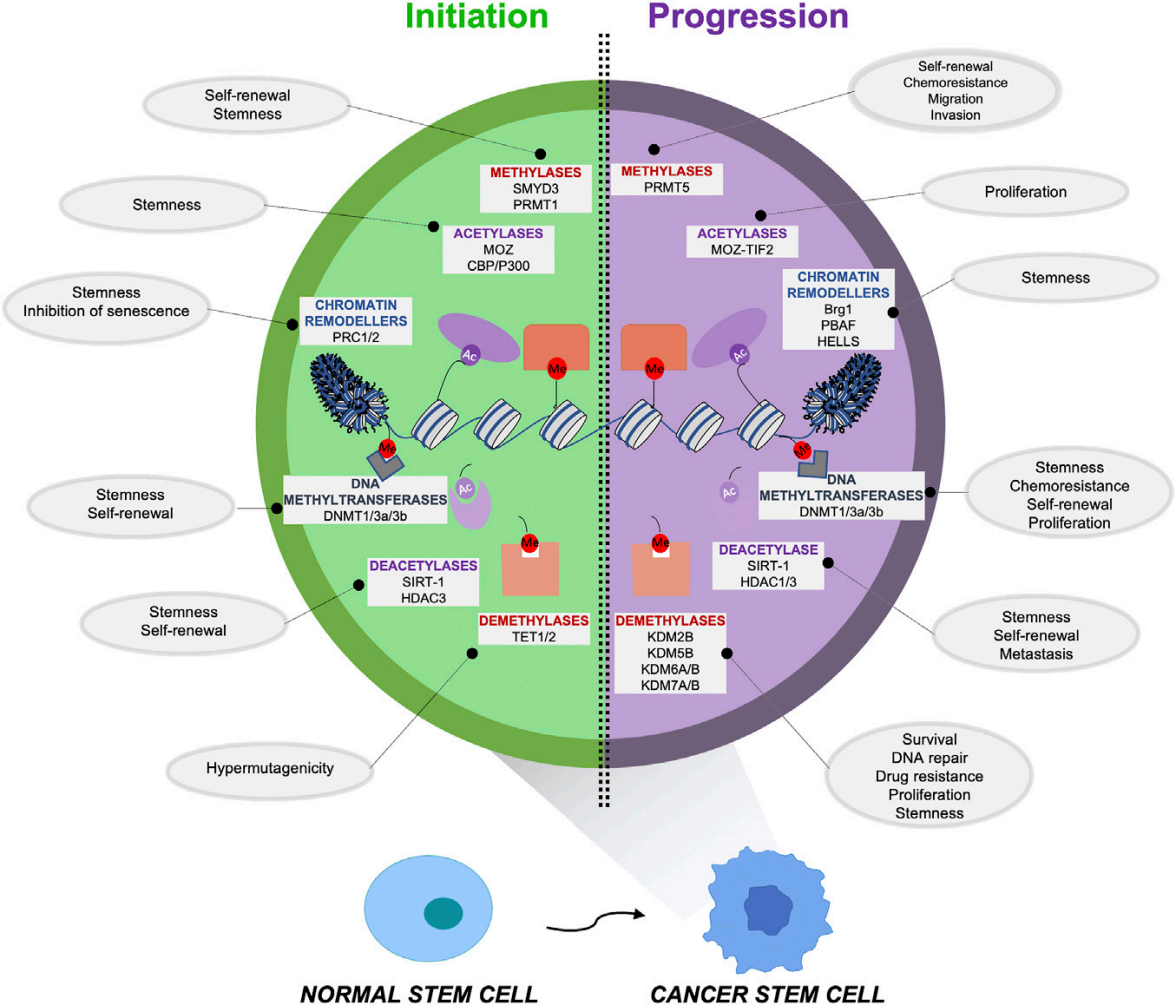
Epigenetic alterations responsible for the initiation and maintenance of cancer stem cells (CSCs). An aberrant epigenetic reprogramming plays a critical role in CSC initiation and maintenance, driving the transformation process of a normal stem cell (NSC) versus a cancer stem cell (CSC). The principal alterations involving the epigenetic regulators that sustain the formation (*left*) and the maintenance (*right*) of this aggressive subpopulation are indicated along with the functions/properties of CSCs that these enzymes are promoting.

In the following paragraphs we will summarize the principal epigenetic mechanisms responsible for CSC initiation, formation and maintenance, which are shown in Figure 1 and described in Tables 1, 2, focusing on the aberrant epigenetic reprogramming driving the transformation process of a NSC to a CSC.

**TABLE 1:** Epigenetic alterations in cancer stem cell (CSC) initiation.

Epigenetic enzymes/LncRNAs	Cancers	Modifications	Functions	References
Methylases				
PRMT1	Leukemia	Upregulation	CSC initiation	Chi et al. (2010)
SMYD3	Gastric cancer	Upregulation	Self renewal, stemness	Wang et al. (2018)
Demethylases				
KDM1A	Prostate and breast cancer, leukemia	Overexpression	CSC initiation	Lim et al. (2010), Crea et al. (2012), Vu et al. (2013)
KDM1B	Glioblastoma	Overexpression	CSC initiation	Hu et al. (2016)
KDM3A	Colorectal cancer	mRNA stabilization	Wnt/β-catenin pathway upregulation	Wang et al. (2019)
Acetylases				
CBP/p300	Leukemia	c-Myb-CBP/p300 interaction	AML1-ETO and MLL fusion-protein upregulation	Pattabiraman et al. (2014)
MOZ	Leukemia	MOZ/TIF2 fusion protein	CSC initiation	Aikawa et al. (2010)
Deacetylases				
HDAC3	Hepatocellular carcinoma	Overexpression	Self renewal, stemness	Liu et al. (2013)
SIRT-1	Colorectal and neuronal cancer	Overexpression	Self renewal, stemness	Chen et al. (2014), Lee et al. (2015a)
Chromatin remodellers				
PRC1	Prostate cancer	Overexpression	Senescence inhibition	Yap et al. (2010)
PRC2	Hematopoietic cancer	Downregulation	CSC initiation	Beerman and Rossi, (2015)
DNA methyltransferases				
DNMT1	Breast cancer	Downregulation	CSC initiation	Pathania et al. (2015)
DNMT3A	Leukemia, myelofibrosis	Downregulation	CSC initiation	Mayle et al. (2015)
DNMT3B	Squamocellular carcinoma	Downregulation	CSC initiation	Rinaldi et al. (2017)
DNA demethylases				
TET1	Non-Hodgking B cell lymphoma	Detection	5-hydroxymethylcytosine loss and DNA hypermethylation	Cimmino et al. (2015)
TET2	Leukemia	Downregulation	Stem cell hypermutagenicity	Pan et al. (2017)
Long non coding RNAs				
HOTAIR	Breast cancer	Overexpression	Self renewal, stemness, CSC initiation	Deng et al. (2017)
HOTTIP	Pancreatic cancer	Overexpression	Self renewal, stemness	Melendez-Zajgla and Maldonado, (2021)
LnchPVT1	Hepatocellular carcinoma	Upregulation	Self renewal, stemness, CSC initiation	Wang et al. (2014)
Linc00617	Breast cancer	Upregulation	Self renewal, stemness, CSC initiation	Li et al. (2017)
MALAT-1	Hepatocellular carcinoma, pancreatic cancer	Upregulation	Self renewal, stemness, CSC initiation	Wang et al. (2015), Zeng et al. (2018)
RNA methyl transferases				
METTL3	Glioblastoma	ncRNA m6A-modification	CSC initiation	Cui et al. (2017)
METTL14	Glioblastoma	ncRNA m6A-modification	Self renewal, stemness, CSC initiation	Cui et al. (2017)

**TABLE 2:** Epigenetic alterations in cancer stem cell (CSC) maintenance.

Epigenetic enzymes/LncRNAs	Cancers	Modifications	Functions	References
Methylases				
PRMT5	Breast cancer	Overexpression	CSC maintenance	Chiang et al. (2017)
Demethylases				
KDM2B	Leukemia, glioblastoma	Overexpression	CSC maintenance, survival and chemoresistance	Frescas et al. (2007), He et al. (2011), Staberg et al. (2018)
KDM6A/B	Glioblastoma	Overexpression	CSC proliferation, drug resistance	Liau et al. (2017)
KDM7A/B	Glioblastoma	NR	CSC survival and DNA repair	Mallm et al. (2020)
Acetylases				
MOZ-TIF2	Leukemia	Overexpression	CSC proliferation	Aikawa et al. (2010)
Deacetylases				
HDAC1	NSCLC	NR	Self renewal and CSC proliferation	Wang et al. (2017)
HDAC3	Hepatocellular carcinoma	NR	Self renewal	Liu et al. (2013)
SIRT-1	Pancreatic cancer	Overexpression	Cancer metastasis and stem cell properties	Leng et al. (2021)
Chromatin remodellers				
Brg1	Colorectal cancer	NR	CSC maintenance	Yoshikawa et al. (2021)
HELLS	Glioblastoma	NR	CSC maintenance	Zhang et al. (2019)
PBAF	Prostate cancer	NR	CSC maintenance	Hagiwara et al. (2021)
DNA methyltransferases				
DNMT1	Hepatocellular carcinoma, breast cancer	NR, overexpression	Self renewal, CSC maintenance	Pathania et al. (2015), Wang et al. (2021)
DNMT3A	NR	NR	CSC proliferation and chemoresistance	Wainwright and Scaffidi, (2017)
DNMT3B	NR, hepatocellular carcinoma	NR	CSC maintenance	Shukla and Meeran, (2014), Lai et al. (2019)
Long non coding RNAs				
H19	Breast cancer, papillary thyroid carcinoma	NR, upregulation, NR, NR	CSC maintenance, proliferation and self renewal	Li et al. (2018), Peng et al. (2018), Peperstraete et al. (2020), Singh et al. (2020)
HOTAIR	Breast, colon and gastric cancer	NR, overexpression, NR	Prometastatic, CSC maintenance, tumor growth	Zhang et al. (2014a), Wang et al. (2016), Deng et al. (2017)
Lnc34a	Colorectal cancer	Overexpression	CSC proliferation	Amirkhah et al. (2019)
MALAT-1	Glioma, breast and pancreatic cancer	Upregulation	CSC proliferation, self renewal, colony formation and invasion	Jiao et al. (2014), Jiao et al. (2015), Han et al. (2016), Zeng et al. (2018)
NORAD	Pancreatic cancer	Upregulation	Self renewal, proliveration and tumorigenesis	Ma et al. (2021)

NSCLC, non small cell lung cancer; NR, not reported.

### Epigenetic changes in tumor initiation: Theories about epigenetic switch of normal stem cells into cancer stem cells

CSCs are characterized by abnormalities in genes that play a pivotal role in normal differentiation. The alterations of these genes mainly involve a hypermethylated profile that characterized CSCs since their premalignant state (Mayle et al., 2015; Pan et al., 2017). The hypermethylated status in these cells is due and maintained by the activity of several epigenetic regulators such as TET1 and TET2, the proto-oncogene IDH1 and IDH2, Polycomb group proteins, that tightly regulate the epigenetic profile of CSCs (Nguyen et al., 2002; Kondo et al., 2003; Cimmino et al., 2015; Turcan et al., 2018). Specifically, the aforementioned epigenetic status is characterized by a bivalent chromatin pattern: histone H3 trimethylated at Lys27 (H3K27me3), a repressive trait, plus dimethylated H3K4 that is active mark (Ohm et al., 2007). On the basis of these observations, several theories regarding tumor initiation were described postulating a crucial role of epigenetics, such as the “Epigenetic Priming model” reported by C. Vicente-Duenas et al. (2018), based on the presence of a cause/effect ratio between the epigenome and genetic alterations. Depending on the SC pre-existing epigenome, the oncogenic hit is a fundamental occurrence that is necessary only in the early stages of tumor initiation reshaping the epigenetic profile of stem cells without inducing any phenotypic modification. This theory is based on numerous observations that suggest that these changes, regarding the epigenetic modification on target genes, remain in a primed state and downstream effects persist even when the oncogene is no longer expressed by the cells during later stages of the tumor pathology. The epigenetic changes thus introduced give rise to an aberrant differentiation program, which ultimately leads to tumor formation. Moreover, by comparing mutational background and epigenetic profiles of healthy vs. tumor tissues, Direna Alonso-Curbelo et al. (2021) demonstrated that tissue damage and oncogene mutations determine a series of epigenetic changes which drive cancer initiation only in tumor tissues while they are not occuring during the physiological processes of tissue regeneration in healthy counterparts. For instance, this epigenetic state early following tissue damage has been reported to induce a dysregulation of survival-associated signaling pathways, thus leading to tumorigenesis in pancreatic cancer patients.

### Epigenetic changes in tumor initiation: Histone modifications and chromatin remodeling

The aim of this paragraph is to highlight the pivotal role of epigenetics, above the variations at the genetic level, in CSC determination and the subsequent tumor initiation. The epigenetic switch, observed in the previously reported studies, is characterized by significant variations in the level of chromatin and genes that regulate the structure and accessibility of chromatin, and in the methylation profile of specific genes. All these changes are fundamental for the understanding of the mechanisms underlying tumor initiation. However, the epigenetic alterations that determine CSC profile are still to be completely defined and represent a challenge in understanding cancer biology. In the recent years, epigenetic mechanisms underlying the switch between NSCs to CSCs have been reported as the basis for CSC cellular plasticity, representing an important focus of study to determine the origins of tumor heterogeneity and the possibility of making reversible the acquisition of a transformed phenotype (Kumar et al., 2022).

Several works, through the use of advanced techniques, focused the attention on both genomic and epigenomic landscape, genes related to the regulation, and the structural alteration of epigenetics. The presence of mutations that give stem cells self-renewal aberrant properties is related to the presence of chromosomal rearrangements. Histone modification represents the most studied epigenetic modification. Histone modification induces structural and functional alteration of cellular phenotype. Histone proteins governed DNA tridimensional structure and each of these modifications is implicated and covers pivotal steps of tumor formation and evolution (Feinberg, 2018). In particular, numerous histone modifications generate epigenetic alterations implicated in tumorigenesis, mainly modulating the gene expression pattern of the SC compartment. Two are the main methylase enzymes involved in the early step of tumorigenesis: PRMT1, especially involved in leukemia SC transformation (Pereira et al., 2010), and SMYD3, that maintains self-renewal and tumorigenicity ability of gastric CSCs (Wang et al., 2018). Likewise, aberrant histone demethylation is responsible for tumor initiation process. KDM3A responsible for demethylation of histone H3 lysine 9, when overexpressed, increases the expression of the Wnt/β-catenin pathway inducing colorectal CSC determination and colorectal cancer tumorigenesis (Wang et al., 2019). Moreover, KDM1A and KDM1B enzymes were found overexpressed in several solid and hematopoietic tumors collaborating in the early step of the tumorigenesis process (Lim et al., 2010; Crea et al., 2012; Vu et al., 2013; Hu et al., 2016).

Of particular importance seems to be the gene KMT2A/MLL encoding for a methyl transferase that regulates the changes at the chromatin level. The formation of fusion proteins, as a result of chromosomal rearrangements at the level of the aforementioned gene, induces the formation of SCs that have “degenerated” into numerous solid tumors and acute myeloid leukemia. Cohesins represent a family of proteins that regulate the definition of three-dimensional structure of chromatin. The degeneration of this family of proteins determines, through significant chromatin changes, the transcriptional profile definition related to neoplastic transformation. Mutation on this protein machinery that govern the tridimensional chromatin structure, have been positively correlated with a degenerated remodeling at the chromatin level and with an increased expression of stemness-related genes in SCs. All these changes are linked to the leukemia SCs arising (Mazumdar et al., 2015) and the degenerated activation of signaling pathways, linked with tumor initiation, such as SFN5-Nanog and SWI/SNF (Wilson and Roberts, 2011). These events have been mostly characterized in liquid tumors, in which CSC model represents a fundamental paradigm. However, many epigenetic alterations have also been observed in solid tumors. Histone modification is strongly represented in cancers that have been found particularly enriched in the CSC compartment. The loss of H3K27me3 induces the expression of stem-related genes such as *SOX9*, *LGR5*, *ASCL2*, *OLFM4* and *EPHB* in colorectal cancer (Lu et al., 2020). Glioblastoma is a tumor specifically studied for the rich stem component, about one-third of glioblastomas of pediatric type are characterized by gain of function at the histone H3 level. In particular, the most altered gene appears to be *H3F3A*, which causes Polycomb repressive complex 2 (PRC2) inhibition. The result is the reduction of the trimethylated form of histone, which makes the chromatin less accessible. This observation clearly illustrates how chromatin plays a fundamental role in defining a transformed profile of CSCs (Wainwright and Scaffidi, 2017).

Likewise, PRC1 induces histone modification by a different mechanism involving monoubiquitination of histone H2A at lysine 119 (H2AK119Ub1). The result of this epigenetic alteration is the inhibition of senescence and the promotion of tumorigenesis through INK4a/ARF repression (Yap et al., 2010). Polycomb group proteins, including PRC1 and PRC2, tightly regulate the methylation profile of CSCs in colorectal and bladder cancers (Nguyen et al., 2002; Kondo et al., 2003). Proteins involved in establishing and maintaining DNA methylation have also been identified as drivers for CSC formation. Compelling evidence suggests that hypermethylation of CpG islands is an early event in cancer development and, in some cases, may occur at SC level contributing to neoplastic transformation. Hypermethylated CpG islands leave a molecular imprinting on cancer cells and can be used as molecular marker to study the evolution of the different epigenetic profiles during tumor growth and progression. DNA methyltransferases (DNMT1, DNMT3A and DNMT3B) and methylcytosine dioxygenases (TET1 and TET2) regulate the methylation status of CpG regions and the occurrence of mutations in these genes may ultimately interfere with protein activity. Several studies show the correlation between the specific amino acid alteration in DNMT3A and a specific gene expression profile (Cancer Genome Atlas Research et al., 2013). For example, most DNMT3A inactivating mutations which are responsible for the malignant transformation of leukemia stem cells (LSCs) have been identified in hematopoietic tumors. Decreased expression of TET proteins have been found in many tumors, suggesting their role in epigenetic stability. Loss of function of TET2 leads to a hypermethylation of target genes and increase the mutation rate of hematopoietic stem cells (HSCs) (Pan et al., 2017). TET1 is mainly expressed in HSCs and plays an important role in SC epigenetic profile maintenance, avoiding DNA hypermethylation. Mutations in *TET1* and the subsequential TET1 protein downregulation lead to 5hmC loss and to malignant transformation of HSCs (Cimmino et al., 2015). IDH1 and IDH2 retain a dehydrogenase activity that has been found altered in many solid tumors resulting in a pro-tumorigenic function. Mutations in proto-oncogene IDH1 and IDH2, are usually found in glioma and acute myeloid leukemia (AML), resulting in a hypermethylated profile that confer a selective advantage to the mutated subpopulation of cells, in term of growth rate and stemness capability (Turcan et al., 2018). Abundant genes that normally suppress tumor growth in normal cells are hypermethylated, such as *RASSF10* in kidney cancer, *SIX3* in glioblastoma, *CDKN2A* and *PTEN* in melanoma. Hypomethylation of several genes may also represent another fundamental step in tumor initiation, including *LY6K* in glioblastoma, *SLC34A2* in papillary thyroid carcinoma (PTC), and *RBBP6* in colorectal cancer.

Of note, the addition of acetyl group on H3/H4 modifying chromatin configuration allows the interaction with transcription factors (Morrison and Thakur, 2021). Multiple enzymes are responsible for catalyzing the addition and removal of acetyl groups, including HATs and HDACs respectively (Lu et al., 2020). The mechanism of histone acetylation on H3/H4 facilitates a tight packaging of chromatin structure (Liu et al., 2017a). For leukemia SCs malignant transformation modification on the acetylation profile is strictly related to the activity of CBP/p300 by the interaction with c-Myb resulting in the induction of acute myeloid leukemia (Pattabiraman et al., 2014). Moreover, leukemia SCs are characterized by the acetylation status of AML1-ETO fusion protein by p300 enzyme (Wang et al., 2011). Another HAT enzyme involved in the transformation process and in the maintenance of malignant phenotype is the fusion protein MOZ-TIF2 increasing the expression of CSF1R (Aikawa et al., 2010). Similarly, HDAC collaborates in the initiation step of several solid tumors. In liver CSCs, HDAC3 is overexpressed compared to normal stem compartment and study conducted by genetic ablation of this enzyme showed its critical role for CSC renewal ability. In fact, HDAC3 knock-out impairs sphere and clone formation and reduces the expression of stem markers (Liu et al., 2013). Furthermore, in colorectal and neural SCs SIRT-1 is required for the maintenance of cell survival and stem phenotype. SIRT-1 deficiency was demonstrated to impair the tumorigenic ability of the aforementioned CSCs (Chen et al., 2014; Lee et al., 2015a).

### Epigenetic changes in cancer initiation: Role of long non-coding RNAs and RNA modifications

LncRNAs are involved in the regulation of stem-like phenotype in several tumors. Many of these ncRNAs play a pivotal role in determining a malignant transformation on progenitor/stem cell tissue, determining the CSCs typical properties (Schwerdtfeger et al., 2021). Lnc-PVT1 is particularly correlated with HCC development and has been found to induce CSC transformation, by stabilizing NOP2 protein (Wang et al., 2014). Linc00617 is a nuclear lncRNA that binds the promoter of *SOX2* and, by epigenetic reprogramming, increases the transcription rate, enhancing self-renewal and promoting stemness in breast cancer cells (Li et al., 2017). Compelling evidence suggests that lncRNAs play a pivotal role in the molecular processes required for determining the stem phenotype (Castro-Oropeza et al., 2018). Moreover, lncRNAs play a key role in the initiation and progression of pancreatic ductal adenocarcinoma (PDAC). Recent studies have shown that these RNAs play a relevant role in the maintenance of CSC phenotype. MALAT-1, a known oncogenic lncRNA, was able to promote stemness and increase the number of pancreatic CSCs, which lead to increased tumorigenicity *in vivo*, probably through the regulation of SOX2/SOX9 (Zeng et al., 2018). In HCC, lncTCF7 is significantly upregulated, increases CSC survival, recruits and activates Wnt pathway components (Wang et al., 2015). Furthermore, HOTAIR is another lncRNA involved in the establishment of CSC properties (Deng et al., 2017). HOTAIR interacts mainly with miRNAs influencing tumor initiation and development (Cantile et al., 2021). In particular, HOTAIR blocks the inhibitory effect of mir-34 on the JAK/STAT pathway, thus activating this crucial stemness-related signaling pathway (Deng et al., 2021). Moreover, HOTTIP is an important epigenetic regulator of CSC phenotype acquisition and stabilization. HOTTIP is a RNA binding protein that activates the HOXA9 transcription factor, which in turn leads to Myc signaling activation and stemness profile determination in PCSCs (Melendez-Zajgla and Maldonado, 2021). HOTTIP drives HOXA9 transcriptional profile even in Leukemia CSCs, activating downstream targets mostly related to Wnt/β-catenin signaling (Luo et al., 2022).

In addition to the role of lncRNAs in the initiation step of tumorigenesis, further epigenetic alterations involve structural and functional RNA alteration through several biochemical-related modification processes (Zhao et al., 2020). The methyltransferases and demethylases recognize, bind and change RNA methylation status influencing the stability, the post-translational modification and the splicing variants reflecting on protein expression (Xie et al., 2020). For example, down-regulation of METTL3 and METTL14 by increasing RNA N6—methyladenosine promotes the survival and the self-renewal ability of glioblastoma SCs thus leading to the acquisition of a transformed phenotype (Cui et al., 2017).

### Role of epigenetic alterations induced by mechanotransduction on cancer stem cells

Mechanotransduction is a biological process through which cells are able to convert mechanical stimuli in biochemical signals. Of note, altered biophysical forces derived from surrounding tumor microenvironment (TME) could promote EMT process and cancer stemness through the alteration of epigenetic signatures. Accordingly, epigenetic enzymes such as histone modifiers have been reported to be regulated by an altered mechanotransduction (Veerasubramanian et al., 2020). The origin and maintenance of CSCs is finely regulated by the mechanotransduction proprieties of extracellular matrix (ECM) through a great variety of cell surface receptors and the physical interaction of CSCs with their surrounding ECMs. Several emerging evidence support the idea that mechanical inputs could epigenetically regulate CSC origin, maintenance and behavior. Tan and colleagues showed that matrix softness regulates CSC maintenance via H3K9 demethylation resulting in increased expression of Sox2 in melanoma CSCs. These findings confirmed the link between matrix softness-induced epigenetic alterations with self-renewal and survival properties of CSCs (Tan et al., 2014). Moreover, the methylation in the promoter region of the oncosuppressor RASSF1A is associated with the constitutive nuclear accumulation of YAP1 and high expression levels of prolyl 4-hydroxylase alpha-2 (P4HA2) which sustain collagen deposition. The elevated collagen deposition induces ECM stiffness, which drives a stem-like reprogramming and promotes the metastatic potential of CSCs in lung adenocarcinoma (Pankova et al., 2019).

## Epigenetic alterations critical for cancer stem cell maintenance

CSCs derive from NSCs in a complex multistep process characterized by both genetic and epigenetic alterations. Epigenetic lesions are a multitude and different in their own nature: they are linked to a modification of the structure and function of the genome or can be associated with limitless uncontrolled cell growth and, generally, to the acquisition of the phenotypic hallmarks of the malignant CSCs. Basically, epigenetic regulations are a primary mechanism which define the SC identity, notably studying CSCs, they show how epigenetically different they are from the healthy counterpart.

Alteration of the epigenetic code, particularly regarding the expression of proteins involved in writing or reading post-translational modifications represent two mechanisms that induce cancer formation. Moreover, carcinogenesis is characterized by epigenetic modifications including change in methylation patterns of cytosines in DNA, modifications of the proteins that bind to DNA, and the nucleosome positioning along DNA. The epigenetic status follows a well-balanced homeostasis in normal cells, but it results strongly altered in many ways in CSCs. The principal epigenetic alterations responsible for CSC maintenance are described in Table 2.

### Alterations in epigenetic regulators as histone modifying enzymes in cancer stem cells

The variety in histone modifications showed a deep complex scenario: Several coexisting histone modifications induce activation, and some of them induce repression. Importantly, these modification patterns are not static but they are a fluid and dynamic changing in cellular context. Furthermore, the activation and repression induced by histone modifications are not necessary mutually exclusive, as announced by ‘‘bivalent domains.’’ The resulting influence that one or more histone modifications display on cell fate is termed ‘‘histone crosstalk,’’ and recent evidence would suggest that crosstalk have a great biological significance, particularly in cancer (Esteller, 2007; Lee et al., 2010). Histone methylation involves mainly the K and R residues and these methylations represent chemical marks that serve as binding sites for histone readers (Stallcup, 2001). K and R methylation can occur on both histones and non-histone proteins. Lysine methylation is directly associated to gene activation or repression. For instance, while histone H3 lysine 4 (H3K4), histone H3 lysine 36 (H3K36), and histone H3 lysine 79 (H3K79) induce gene activation, histone H3 lysine 9 (H3K9) and histone H3 lysine 27 (H3K27) lead to gene repression. In many cancers, epigenetic modifications are associated not only with the malignant transformation of NSCs into CSCs, but also influence their stemness phenotype and promote tumor progression. Accordingly, PRMT5 is an arginine methyltransferase, which guides both *in vitro* and *in vivo* breast CSCs (BCSCs) proliferation and self-renewal via the transcription factor FOXP1. Mechanistically, PRMT5 is recruited on the FOXP1 promoter and catalyzes Histone H3 R2 Dimethyl Symmetric (H3R2me2s), which in turn induce the recruitment of SET1 and favors the H3K4me3 with subsequent stemness genes expression (Chiang et al., 2017). Similarly, brother of the regulator of the imprinted site (BORIS) regulates CSC-like properties in liver cancer via OCT4 expression. Particularly, BORIS overexpression facilitates its binding on the OCT4 promoter and it has been associated with high levels of H3K4me2 (Liu et al., 2017b). Interestingly, methylation alteration was found in inflammatory disease model, particularly EZH2 catalyzes trimethylation of histone 3 lysine 27 (H3K27me3) is critical for ameliorate the intestinal immune regulation during inflammatory bowel disease (IBD) (Zhou et al., 2019). Accordingly, Kelly et al. (2018) identified a gene signature showing iperactivation of pathways involving immunoregulation, cell survival and metabolism associated to altered levels of H3K4me3, that correlated with worst prognosis in IBD patients.

The initial idea that histone lysine methylation was a highly durable, static modification has now been confuted by the identification of eight classes of lysine demethylases (KDM1-8). Depending on which lysine residue is modified, KDMs can regulate transcriptionally, by activating or repressing, both oncogenes and tumor suppressors. The KDM7 subfamily has catalytic activity at lysine residues on histone tails. KDM7A and KDM7B demethylase H3K9me2/me1, H3K27me2/me1 and H4K20me1, while KDM7C only demethylases H3K9me2 (Lee et al., 2015b; Pappa et al., 2019). KDM7A maintains low levels of H3K9 and H3K27 methylation by guaranteeing viability of glioblastoma SCs (Mallm et al., 2020). Accordingly, some studies show how KDM2B is characterized by demethylase activity at H3K4me3 KDM2B has an important role in leukemia SC maintenance and proliferation of glioblastoma SCs (Frescas et al., 2007; He et al., 2011). Of note, the knockdown of KDM2B in glioblastoma cells decreases the levels of H3K36me2, reduces the number of proliferating cells and increase DNA damage accumulation, supporting KDM2B role in glioblastoma SC maintenance (Staberg et al., 2018). The histone demethylases KDM6A/B are highly expressed in glioblastoma SCs and *via* NOTCH pathway induce plasticity in CSCs contributing to tumor progression and relapse (Liau et al., 2017).

The lysine residue acetylation represents a histone modification involved in DNA repair machinery, transcription and chromatin structure modeling. Biologically, acetylation induces a neutral charge on lysine’s positive residues by leading to a reduction of electrostatic interaction between positive histones and negatively charged DNA. For this reason, histone acetylation is linked to a more ‘‘open’’ chromatin conformation. Acetylation is regulated by the competing activities of two families of epigenetic enzymes, the histone lysine acetyltransferases (KATs) and HDACs. Specifically, KATs are divided in two groups: 1) type-B, which are predominantly cytoplasmic, and 2) type-A, which are primarily nuclear. The expression of KATs and HDACs impairs the structure and integrity of the genome of NSCs, so these two groups of histone modifiers drive CSC transformation in several cancers.

The constitutively expressed acetyltransferase fusion protein MOZ-TIF2 is able to interact with transcription factor PU.1 by inducing the transcriptional activation of CSF1R. In an *in vivo* model, PU.1\MOZ-TIF2 interaction drives the upregulation of CSF1R and it leads to maintenance of LSCs (Aikawa et al., 2010). Upon chronic Cr(VI) exposure, lung cancer cells display a glycolytic shift, which is directly associated with an upregulation of the proto oncogene c-Myc expression. Moreover, this glycolytic shift in Cr(VI)-transformed cells promotes an increased production of acetyl-CoA and induces histone acetylation, thus enhancing CSC-like properties and their tumorigenic capacity (Clementino et al., 2020).

A pro-tumorigenic role of HDACs has been demonstrated in a model of non-small cell lung cancer (NSCLC), resistant to cisplatin (CDDP)-based therapy. *In vivo* studies confirmed that CDDP resistant tumors display high expression levels of CSC-associated transcription factors. CDDP-enriched CSCs show an aberrant activation of the C/EBP-β/TRIB1/HDAC1/p53 axis. Specifically, Wang et al. (2017) hypothesized that C/EBP-β induces the transcription of the protein kinase TRIB1, which cooperates with HDAC1 promoting p53 acetylation and activation. This axis is involved in CSC enrichment and chemotherapy resistance induced by CDDP in NSCLC. Several studies showed high expression levels of HDACs in CSCs. In particular, increased HDAC3 levels has been observed in liver CSCs and positively correlated with Nanog and CD133 expression levels (Liu et al., 2013). The HDAC SIRT-1 is highly overexpressed in CD133^+^ colorectal stem-like cells (Chen et al., 2014). Moreover, SIRT-1 was found to maintain, in cooperation with CRL4B complex, the stemness features of pancreatic CSCs (Leng et al., 2021).

### Alterations in epigenetic regulators as chromatin remodelers in cancer stem cells

Basic biological events such as transcription, replication and DNA repair depend on DNA accessibility to the enzyme complex implicated in each process. ATP-dependent chromatin remodelers are able to define DNA accessibility by acting on nucleosomes for repositioning, ejecting, or modifying their structure. Eukaryotic cells are characterized by four families of chromatin remodelers, which are classified on the similarities and differences of the ATPase subunits, including SWI/SNF, imitation switch (ISWI), chromodomain helicase DNA-binding (CHD), and INOsitol requiring 80 (INO80). The epigenetic deregulation can derive from chromatin remodeling that results altered in various ways in cancer: redistribution or mistargeting; down or over expression of subunits; loss-of-function mutations in SWI/SNF-subfamily remodelers which is linked to reduction of DNA accessibility at promoters and enhancers of tumor-suppressor or other genes; or gain-of-function mutations in SWI/SNF-subfamily remodelers which cause high dynamic nucleosome mobility and DNA accessibility at oncogenes or genome-wide. This scenario is frequently observed in CSCs and other diseases. For example, Brg1 is a chromatin-remodeling regulator for maintenance of intestinal CSCs. Specifically, Brg1 plays a crucial role in intestinal CSCs in mice by affecting apoptosis and improving cell survival and SC subpopulation in human colorectal cancer cells (Yoshikawa et al., 2021). Similarly it has been demonstrated that MUC1-C, an oncogenic protein, interacts with SWI\SNF family complex PBAF by balancing ROS levels and pluripotency gene expression in prostate CSCs (Hagiwara et al., 2021). Therefore in silico analysis, the chromatin remodeler HELLS was able to form a complex with the transcription factors Myc and E2F3 by promoting stemness in glioblastoma cells (Zhang et al., 2019). Besides, it has been found how the SWI/SNF complex activates transcriptionally the oncogenes AR/FOXA1 expression in prostate cancer (Xiao et al., 2022).

Taken together this data indicate that both histone modifications and chromatin remodeling alter the gene expression of oncogenes and/or tumour suppressor genes thus affecting genome integrity and unbalancing NSCs homeostasis in favor of CSCs transformation.

### DNA methylation in cancer stem cells maintenance

DNA methylation is a covalent chromatin modification regulating genome stability and gene expression. The lack of DNA methylation regulation mechanisms causes several diseases, including cancer. CSCs present an aberrant DNA methylation, mainly occurring in CpG islands. Accordingly, several tumor suppressor genes (TSGs) are hypermethylated in cancer and, for this reason, silenced by promoting cancer progression. Changing of DNA methylation pattern (hypomethylation or hypermethylation) in cancer is often associated with an aberrant expression of DNMTs (1, 3A, and 3B). Specifically, DNMT family has a crucial role for maintaining the CSC statement (Wainwright and Scaffidi, 2017). It has been shown that DNMT1 ablation could decrease proliferation and tumorigenesis in lung CSCs, besides it leads to the extinguished of CSCs from tumor bulk by enhancing apoptosis and differentiation (Toh et al., 2017; Gao et al., 2018). Accordingly, DNMT1 is able to regulate BEX1 in liver cancer, by controlling self-renewal and maintenance of liver CSCs via Wnt/β-catenin signaling pathway activation (Wang et al., 2021). It has been found that DNMT1 expression is overexpressed in breast cancer, and mammary gland-specific DNMT1 deletion protects *in vivo* mouse model from breast cancer tumorigenesis by reducing BCSC pool. Through genome-scale methylation approach, it has been identified ISL1 as a direct DNMT1 methylation target. Specifically, ISL1 is hypermethylated and downregulated in breast tumors and CSCs subpopulation (Pathania et al., 2015).

DNMT3B is involved in the increasing methylation of CSCs guaranteeing an undifferentiated state (Shukla and Meeran, 2014). Therefore, mutations in *DNMT3A* also induce the maintenance of CSC subpopulation in cancers. These *DNMT3A* mutations directly activate CSC proliferation pathways and increase chemoresistance (Wainwright and Scaffidi, 2017). In accordance, DNA methylation controls the expression of surface markers of CSCs such as CD44 and CD133 for CSC maintenance, as well as ABC transporters, involving drug efflux into CSCs promoting chemotherapy resistance (Crea et al., 2009). Moreover, it has been found that DNMT3B/OCT4 axis expression induces sorafenib resistance and stem-like properties in HCC via IL-6/STAT3 pathway regulation (Lai et al., 2019).

A deeper analysis of DNA methylation patterns in CSC population is crucial for a better understanding of the molecular mechanisms CSC-driven associated to tumor relapse and poor prognosis, and will lead to the identification of more specific DNA methylation inhibitors.

### Long non-coding RNA role in cancer stem cell maintenance

NcRNAs are a subgroup of RNAs that do not translate into protein but control gene expression at the post-transcriptional level, by considering them as an important epigenetic regulator. Particularly, lncRNAs (ncRNAs over 200 nucleotides in length) have a critical function in maintaining CSC populations through stemness genes regulations and, generally, by activating pathways related to SCs (Chen et al., 2017).

The expression of HOTAIR is closely linked with advanced tumor stage, metastasis, and poor prognosis in a variety of human cancers (Min et al., 2017). HOTAIR’ pro-metastatic role is developed by the targeting of polycomb repressive complex 2 (PRC2) and by the downregulation of metastasis repressor genes (Deng et al., 2017). Moreover, HOTAIR is upregulated in CSC populations of breast and colon cancer. Particularly, in colon CSC subpopulation (CD133^+^/CD44^+^) shows overexpressed levels of HOTAIR, suggesting that HOTAIR regulates the acquisition of stemness. In breast cancer, HOTAIR is able to suppress the tumor inhibitor miR-7, by upregulating the expression levels of c-Myc, TWIST and miR-9 and maintaining the BCSC pool (Zhang et al., 2014a). In addition, HOTAIR was found to unpair the association of P300, CREB and RNA pol II to the SETD2 promoter region by promoting the growth of human liver CSC (Wang et al., 2016).

Additionally, Lnc34a has been found overexpressed in colorectal CSCs. Mechanistically, Lnc34a recruits both DNMT3A and HDAC1 on MIR34A promoter, by inducing the methylation and deacetylation of the promoter. Both the epigenetic perturbations blocked the *MIR34A* gene expression and enhanced CSC proliferation (Amirkhah et al., 2019).

MALAT-1 is a lncRNA upregulated in glioma SCs, BCSCs and PCSCs. It enhances CSC properties, such as proliferation, self-renewal, colony formation and invasion *in vitro* (Jiao et al., 2014; Jiao et al., 2015; Han et al., 2016; Zeng et al., 2018; Amirkhah et al., 2019). Mechanistically, MALAT-1 may represent a molecular sponge for miRNAs. Particularly, MALAT-1 binds miR-200c, which induces an upregulation of ZEB1, an important epithelial–mesenchymal transition (EMT) transcription factor (Korpal et al., 2008; Pa et al., 2017).

Similarly, the lncRNA NORAD was found to be upregulated in pancreatic cancer tissues and cells by acting as a molecular sponge. Accordingly, the lncRNA NORAD could selectively bound to miR-202-5p, by inducing the expression of the miR202-5p target gene *ANP32E*, which is linked to self-renewal of PCSCs and proliferation *in vitro*, as well as enhancing tumorigenesis of PCSCs *in vivo* (Ma et al., 2021).

The lncRNA H19 controls the maintenance of the CSC pool. Accordingly, microarray analysis showed that high levels of H19 are positively correlated with the overexpression of a cluster of transcriptional factors stemness-related such as Sox2, Oct4, and Nanog in acute lymphoblastic leukemia cells (Singh et al., 2020). In addition, BCSCs (ALDH1A1+; CD44+/CD24−) showed high H19 expression levels, induced by miR-675, supporting a role for both H19 and miR-675 in the enrichment of BCSC compartment (Peperstraete et al., 2020). LncRNA H19 plays a role in regulating CSC maintenance also through its function as a miRNA sponge. Of note, it has been found that H19 and LIN28 cooperate in promoting CSC self-renewal. Increasing levels of LIN28 are regulated by H19 through the sponging of miR-196b (Ren et al., 2018). Similarly, H19 can also sponge miR-3126-5p to increase the expression of ERβ receptor in PTC by promoting CSC-like properties in PTC (Li et al., 2018). Furthermore, H19 regulates miR-let-7 by controlling self-renewal in BCSCs (Peng et al., 2018).

The novel studies of lncRNA biology in human disorders are opening new scenarios for the use of lncRNA as disease biomarkers and/or therapeutic targets. However, to increase our current knowledge, more methodological improvements are necessary in order to study lncRNA structure and unravel the spatial, developmental specificities, and biological networking of lncRNAs.

### Epigenetic reprogramming during inflammation in cancer stem cells

Chronic inflammation has been correlated to several cancers and it contributes to different steps during carcinogenesis (Di Franco et al., 2021). A plethora of inflammatory cytokines, interleukins, interferons, transforming growth factors, chemokines, and adhesion molecules have been associated with chronic inflammation. These inflammatory mediators are directly reported to regulate aberrantly the transcriptomic and the epigenetic machinery, particularly DNA methylation and histone modifications in cancers, driving the pathogenesis of tumor and by fueling CSC pool. For instance, TGF-β regulates DNTMs transcription and activity, leading to radical changes in DNA methylation for the acquisition of stemness phenotype by ovarian cancer cells (Cardenas et al., 2014). Similarly, TGFβ regulates the recruitment of epigenetic enzymes such as DNMTs and HMTs (EHMT2 and SUV39H1) to the *CDH1* gene promoter by inducing endothelial cell transformation, which seems to contribute to the development of BCSCs (David and Massague, 2018). The TGFβ pathway through Smad2/3 induces EMT, which is directly linked to CSC generation. Specifically, the arginine methylation of Smad7 by PRMT1 induces TGFβ-induced EMT and CSC generation (Katsuno et al., 2018). An alternative important mechanism, which involves the TGFβ/Smad-induced EMT in CSC generation, is characterized by two double-negative feedback loops: Zeb/miR-200 and Snail-miR-34. The two double negative loops drive both EMT and CSC generation (Brabletz and Brabletz, 2010; Siemens et al., 2011; Zhang et al., 2014b). Therefore, the Akt/miR-200/E-cadherin axis controlled by TGFβ pathway induces both EMT in mammary epithelial cells and CSC generation (Iliopoulos et al., 2009).

IL-6 mediated inflammation regulates cancer cell stemness. Mechanistically, it exists a specific loop in which p53 deletion causes demethylation of the IL-6 promoter by activating IL-6 signaling. Subsequently, the hyperactivation of IL-6 induces the overexpression of DNMT1, which, in turn, methylates the promoter of the p53 gene by initiating IL-6\p53\DNMT1 autocrine loop (Hodge et al., 2005; D'Anello et al., 2010; Liu et al., 2015). This autocrine loop drives cancer cells to a stem-like phenotype acquisition via epigenetic reprogramming (D'Anello et al., 2010; Liu et al., 2015). Similarly, IL-6 induces hypermethylation of the promoter of the miR142-3p gene by repressing its expression and promoting cell stemness and invasiveness in glioblastoma (Chiou et al., 2013). Interestingly, IL-6 has also a critical role in the conversion of non-CSCs into CSCs (Iliopoulos et al., 2011). Moreover, the IL-6/STAT3/PTEN/NF-κB inflammatory axis is preferentially activated in CD44^+^CD24^−^ stem-like breast cancer cells compared with other tumor cell types such as cancer associated fibroblasts and inflammatory cells (Iliopoulos et al., 2011; Marotta et al., 2011).

## Epigenetic therapy using stem cells for regenerative medicine

A clear understanding of epigenetic processes could be crucial for the full control of SCs and their use in regenerative medicine. Excellent progress in the biomedical field leads to the use of SCs for the regeneration of organs and tissue that have lost their physiologic functions and mechanisms. Epigenetic mechanisms may drive the cellular reprogramming to convert somatic cells into induced pluripotent stem cells (iPSCs), which can be directed to differentiate in specific cell types.

The employment of SCs for patients that are affected by diseases such as type 1 diabetes mellitus, hematological malignancies or cancer represents a new effective therapeutic strategy. Although pharmacological therapy in type 1 diabetes mellitus was excellent to restore the chronic disease and avoid complications, the possibility to re-generate new islets by using embryonic SCs (ESCs) is a promising approach (Chen et al., 2020). Legoy et al. (2020) demonstrated that the human- iPSCs (h-iPSCs) are able to generate insulin-producing β-cells. Proteomic analysis of the transplanted cells revealed that *in vivo* microenvironment is predominant to drive to islet profile, selecting specific hormones through regulation of epigenetic factors and generating human pancreatic progenitors. This study highlighted the capacity of hiPSCs, under particular conditions, to differentiate in β-cells.

**FIGURE 2:**
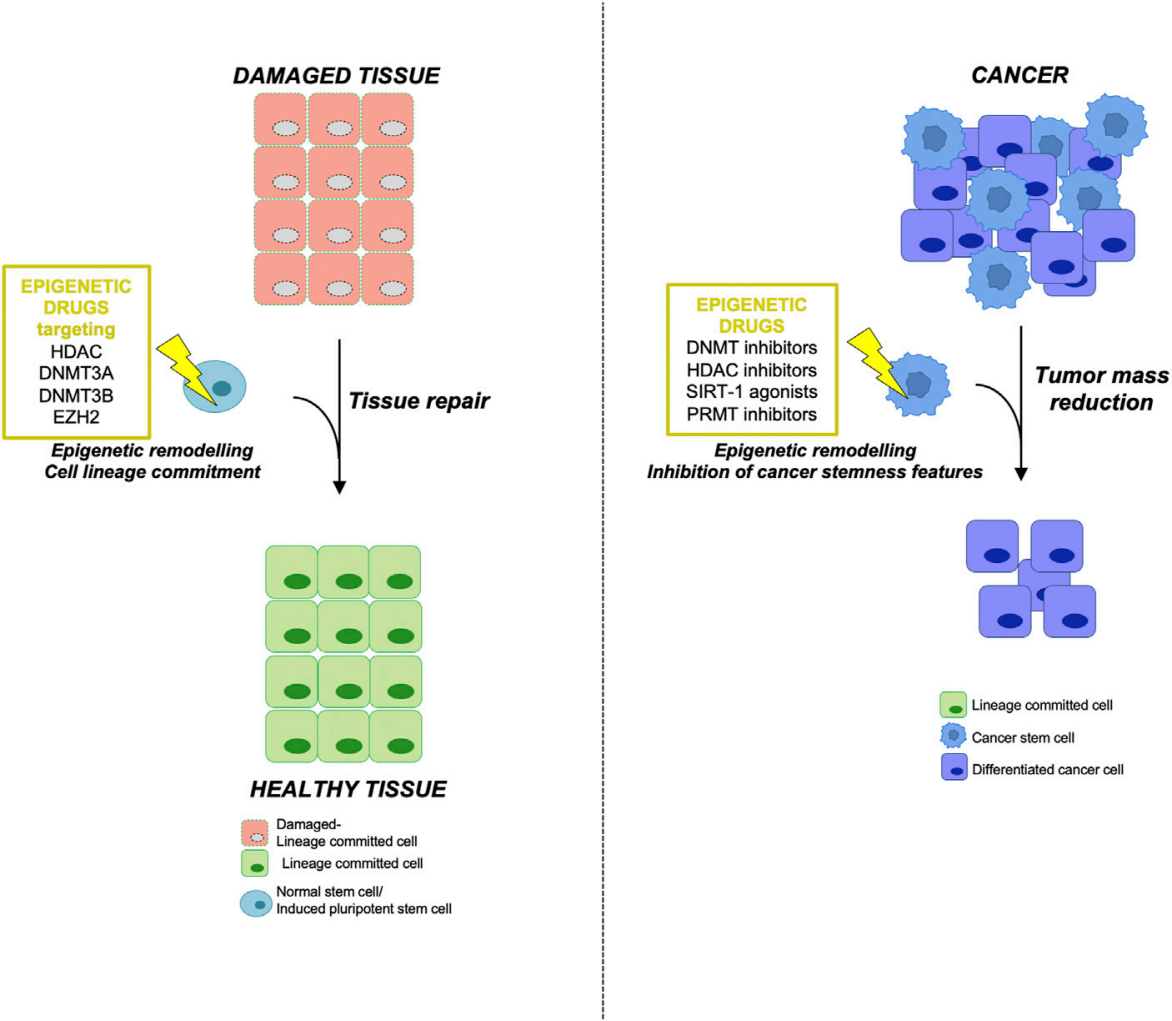
Epigenetic therapy using NSCs in regenerative medicine and targeting CSCs in tumors (*left*) Epigenetically engineered adult normal stem cells (NSCs) or induced pluripotent stem cells (iPSCs) represent an innovative and promising tool in regenerative medicine. Adult stem cells deriving from damaged tissue or iPSCs could be reprogrammed using the indicated epigenetic drugs in order to obtain a specific lineage commitment, thus allowing tissue repair and regeneration. (*right*) On the other hand, the use of epigenetic inhibitors in cancer therapy targeting the aberrant epigenetic modifications responsible for CSC initiation and maintenance may lead to a reduction of tumor mass by inhibiting the stemness features of CSC subpopulation.

Although the use of SCs is wrapped by various problems such as the isolation and expansion *in vitro* along with ethical issues, there are promising and compelling results on the use of SCs as a possible therapeutic approach for regenerative medicine (Figure 2).

Regenerative medicine is used to repair or replace damaged human tissue by engineering adult and embryonic SCs. The concepts of regenerative medicine hold the potential to help in healing previously irreparable tissues or correcting genetic defects responsible for diseases. Bioengineering *in vitro* human SCs, towards evaluating genetic variation responsible for the disease and identifying new targets for therapy, strikes a new direction for regenerative medicine as a potential approach for cell-based therapies (Bailey et al., 2014; Mendelson and Frenette, 2014). Epigenetic control of gene expression is a trait heritable during cell divisions and has emerged as a key mechanism defining the cell lineage commitment and influencing the evolution of many diseases (Choi and Friso, 2010; Hirabayashi and Gotoh, 2010). Adult and embryonic SCs represent an excellent tool to understand human development and organogenesis and may be used to treat several diseases by manipulation of environmental signals and intracellular pathways influencing cell proliferation, self-renewal ability, and cell lineage differentiation (Audet, 2004). The phenotypic and functional changes associated with the commitment of SCs into progenitor precursors and then to terminally differentiated cells are the results of remarkable changes in gene expression patterns. To a large extent, a unique epigenetic program orchestrates the promotion and maintenance of the gene expression profile during differentiation involving the silencing of self-renewal genes and the activation of cell type-specific genes (Wu and Sun, 2006).

Engineered SCs could be used to favour tissue repair upon several pathological conditions and for treating multiple inherited and degenerative diseases such as hematopoietic and immune system disorders, diabetes, Parkinson’s and Alzheimer’s diseases. Moreover, bioengineered SCs are exploited also as a cell-based therapy for the treatment of aggressive and recurrent cancers (Mimeault et al., 2007). Yamanaka and colleagues demonstrated that terminally differentiated cells could undergo unnatural conversion into iPSCs (Takahashi and Yamanaka, 2006). Oct4, Sox2, Klf4, and cMyc, ectopically expressed in terminally differentiated cells, cooperate with PRC2 proteins silencing lineage-specific genes and inducing reprogramming of cell identity and function (Pereira et al., 2010). A characteristic trait of this epigenetic reprogramming is the methylation of H3 histone (Mansour et al., 2012). This discovery breaks up the paradigm according to the cellular destiny that may only follow the natural lineage direction recapitulating embryonic development (Cherry and Daley, 2012). Moreover, iPSCs are easier to obtain from patients compared to SCs and for this reason represent an important source for regenerative medicine applications.

Epigenetic mechanisms cooperate in defining the fate of every cell. DNA methylation, histone modifications, and ncRNAs are responsible for gene expression control during embryonic development, and physiological and pathological processes in adults (Wu and Sun, 2006). Engineering SCs or iPSCs, by inducing an epigenetic reprogramming and modifying downstream gene regulatory networks, is a strategy exploited in regenerative medicine to drive cell destiny. Many epigenetic labels have been found as character traits of many neoplastic and pathological tissue. Furthermore, different epigenetics characteristics are strongly correlated to the differentiation status of a particular tissue or cells. Recent publications highlighted the different roles of each specific DNMT during the transition of SCs from quiescence to proliferating state up to differentiation status (Naito et al., 2016). For example, early-stage steps in normal hematopoietic development are associated with hypermethylation and the loss of function of the methylation-responsible enzymes (Gore and Weinstein, 2016).

Interestingly, histone modification, especially the inactivation of the EZH2 subunit, impairs the self-renewal ability of SCs reducing their regenerative potential (Juan et al., 2011). In many adult tissues, epigenetic modifications regulate cellular plasticity in committed cells, contributing to tissue repair machinery. As reported in the literature, differentiated hepatocytes, in response to tissue damage undergo a rewiring of genomic methylome/hydroxymethylome landscapes reverting lineage-committed and contributing to the regeneration of liver parenchyma (Aloia et al., 2019). Epigenetic modifications influence cell signaling but can be tightly controlled by intra and extracellular factors. As an example, Notch signaling is precisely regulated in a time- and space-restricted manner regulating the quiescence status of stellate cells that represent the staminal compartment of muscle tissue (Bjornson et al., 2012). The myogenesis process is characterized by high cellular plasticity and is strictly controlled by the epigenetic process. Hypomethylation and/or hydroxymethylation of some intragenic or intergenic regions of Notch receptors interfere with the terminal differentiation of myoblasts into mature myofibers.

DNA methylation is one of the major repressive systems for the muscle gene. DNMT inhibitor delivery has been proved to be an alternative strategy for patients affected by dystrophic muscles restoring the regenerative ability of stellate cells (Sincennes et al., 2016). Moreover, epigenetic mechanisms are the main regulator of gene expression patterns during the differentiation of MSCs (Ghorbaninejad et al., 2020). Modulation of DNMT3A and DNMT3B activity influences the whole process of underlying bone tissue development. Mouse depleted for DNMT3B die during embryonic development since it was demonstrated to be necessary for proper lengthening and mineralization of both axial and appendicular bones. DMNT3B is reactivated during the regeneration of bone tissue decreasing at the final stages (Xu et al., 2018). Moreover, the alteration of the methylation status in immune cells is involved in the pathogenesis of autoimmune disease. The association between the methylation status of methylation-sensitive genes and the development of the immune thrombocytopenia (ITP) is currently under evaluation in a clinical trial (NCT04100876). In particular, many single nucleotide polymorphisms (SNPs) in the DNMT3A and in the DNMT3B gene may influence catalytic activity of these enzymes and may be used as prognostic markers (NCT04100876).

The growing interest in the role of epigenetics as cause-and/or-effect of several diseases and as a key regulator of human tissue organogenesis and differentiation has led to intensifying research for the development of new more specific drugs. Targeting epigenetic machinery appears to be a promising therapeutic strategy for several diseases such as neurodegenerative disorders and oncology pathologies. One of the most used epigenetic modulators in the clinic for the treatment of epilepsy patients and bipolar disorders is valproic acid (VPA), a HDAC inhibitor that has been prescribed for several years without any severe side effects (Amitai et al., 2015). VPA enhances the activity of the inhibitory transmitter gamma-aminobutyric acid (GABA) reducing its degradation and promoting its synthesis. Nowadays, several epigenetic drugs are under pre-clinical and clinical evaluation (NCT01021449; NCT04608448; NCT02284477). Epigenetic drugs act on chromatin structure by the inhibition of DNMTs and HDACs influencing the time and space of gene expression (Rodriguez-Paredes and Esteller, 2011; Altucci and Rots, 2016). In orthopedic regenerative medicine, epigenetic drugs represent a promising therapeutic approach (van Wijnen and Westendorf, 2019). Several EZH2 inhibitors such as GSK126, UNC 1999, and EPZ005687, inhibit osteoclastogenesis and promote bone regeneration, and are currently in clinical trials to treat patients with osteoarthritis (van Wijnen and Westendorf, 2019; Ball et al., 2022).

## Epigenetic drugs in clinical trial targeting the epigenetic modifications sustaining cancer stem cells

Epigenetics is crucial for the organism’s development and is highly responsive to environmental cues (Baylin and Jones, 2011). To date, a link between the microenvironment (diet, exposure to chemicals) and epigenetic alterations associated with pathological conditions has not been assessed. In this regard, epigenetic alterations become attractive targets for the treatment of different cancers and diseases. Moreover, epigenetic modifications, due to their reversible nature, represent potential biomarkers useful for clinical purposes (Nebbioso et al., 2012).

A better knowledge of the exact pattern of epigenetic modifications could help clinicians to identify a personalized therapy. In glioma patients, in fact, the expression of O6-methylguanine-DNA methyltransferase (MGMT), an enzyme involved in alkylating agent-induced DNA damage repair, is predictive of response to the treatment with carmustine and temozolomide. Indeed, in presence of a hypermethylated *MGMT* promoter, the ability to repair the damage induced by alkylating agents is consequently decreased, resulting in an increased sensitivity to these chemotherapeutic drugs (Yu et al., 2019). Heritable or environmental-induced epigenetic abnormalities are associated with several diseases, and the differences between healthy and pathological tissues allowed the identification of diagnostic and/or prognostic biomarkers. In this regard, FDA approved an early colorectal cancer-screening program, based on the identification of specific DNA methylation patterns of selected gene promoters in non-invasive tissue (e.g., vimentin, or BMP3, septin and NDRG4). Moreover, DNA methylation at the promoter of brain-derived neurotrophic factor (BDNF) is currently being tested for the treatment of autism or depression (Delgado-Morales et al., 2017). In addition to DNA methylation altered profiles, aberrant epigenetic modifications can derive from somatic mutations occurring in the genes codifying for histone-modifying enzymes and/or chromatin remodelers, altering their expression levels and activity, thus predisposing to cancer and diseases (Han et al., 2019). Importantly, the different families of epigenetic enzymes such as readers, writers, and erasers, can be targeted by epigenetic probes/inhibitors to treat different cancer types.

In the following paragraphs we will revise the epigenetic inhibitors in clinical trial for several cancers focusing on the compounds targeting the epigenetic modifications sustaining the initiation and maintenance of CSCs (Figure 2; Table 3).

**TABLE 3:** Epigenetic inhibitors targeting epigenetic alterations of cancer stem cells (CSCs).

Drugs	Target	Cancer	References/NTC
DNMT inhibitors			
Azacitydine	DNMT1	AML, MDS	NCT01074047
Decitabine	DNMT1	AML, MDS	NCT00043381, NCT00866073
Disulfiram	DNMT1	Recurrent glioblastoma, prostate and metastatic breast cancer	Lin et al. (2011), NCT03323346, NCT02678975
Hydralazine	DNMT1	Ovarian, testis, lung, breast and cervix cancers	NCT00404508
EGCG	DNMT1 and DNMT3A/B	Colorectal, esophageal and prostate cancers	Fang et al. (2003)
Guadecitabine (SGI-110)	DNMT1/3	HCC, AML, MDS, CMML	NCT01752933, NCT02348489, NCT02920008, NCT02907359
SGI-1027	DNMT1 and DNMT3A/B	Histiocytic lymphoma, Burkitt’s lymphoma, breast and prostate cancer	Datta et al. (2009)
HDAC inhibitors			
Tefinostat	Pan HDAC	CTCL, PTCL	NCT02759601
Vorinostat (SAHA)	Pan HDAC	Breast cancer	NCT 04190056
Romidepsin	Class I HDAC	HCC	Barbarotta and Hurley, (2015)
SIRT-1 agonists			
EX-527 (SEN0014196 or selisistat)	SIRT-1	HCC, brast cancer	Gollavilli et al. (2015), Ceballos et al. (2018)
Nicotinammide	SIRT-1	CLL, NMSC	NCT04844528
suramin	SIRT-1	NSCLC, hormone-Refractory Prostate, adrenocortical cancer	NCT01038752, NCT00002723, NCT00002921
PRMT inhibitors			
GSK3326595	PMRT5	Solid tumors, non-Hodgkin’s lymphoma	NCT02783300
LLY-283	PMRT5	Breast, gastric, lung, skin, ovarian and hematological cancers	Bonday et al. (2018)

CSC, cancer stem cell; NTC, national clinical trial; AML, acute myeloid leukemia; MDS, myelodysplastic syndrome; HCC, hepatocellular carcinoma; CMML, chronic myelomonocytic leukemia; CTLT, cutaneous T-cell lymphoma; PTCL, peripheral T-cell lymphoma; NSCLC, non small cell lung cancer; CLL, chronic lymphocytic leukemia; NMSC, non-melanoma skin cancer.

### DNA methyltransferase inhibitors

An aberrant methylation status has been associated with tumorigenesis, and metastasis formation. Moreover, altered DNA methylation patterns have been correlated with poor prognosis and therapy resistance in cancer patients (Romero-Garcia et al., 2020). Different DNA methylation patterns were observed in colon, prostate, lung, ovarian and breast cancers, particularly represented in NSCs versus CSCs (Pechalrieu et al., 2017).

DNMT inhibitors can reprogram cancer cells by inducing cell death and growth arrest. Decitabine (5-aza-2′-deoxycytidine) and azacitydine (5-azacytidine) are nucleoside inhibitors used for the treatment of AML and myelodysplastic syndrome (MDS) (NCT01074047, NCT00043381, NCT00866073). The FDA-approved guadecitabine, 5-Fluoro-2′-deoxycytidine (FdCyd), and zebularine belong to the next-generation hypomethylating agents (Nepali and Liou, 2021). In particular, guadecitabine (known as SGI-110) promotes enhanced uptake of the active metabolite, decitabine, into the DNA of quickly dividing cancer cells, and is resistant to the action of cytidine deaminase (Issa et al., 2015). SGI-110 is currently being tested for the treatment of advanced HCC (NCT 01752933) and the treatment of AML, musculoskeletal disorders (MSDs) and chronic myelomonocytic leukemia (CMML) in patients refractory to standard therapies (Global phase 3 ASTRAL-1, NCT02348489, ASTRAL-2 NCT02920008, and ASTRAL-3 study NCT02907359). The use of nucleoside DNMT inhibitors is associated with genomic instability and risk of mutagenicity, for this reason, non-nucleoside analogs were developed. Mainly, these inhibitors consist of small molecule agents derived from natural and chemical sources, targeting specifically the catalytic region of the methyltransferases. Among the natural non-nucleoside DNMT inhibitors, EGCG (epigallocatechin-3 gallate), a polyphenol derived from green tea demethylates the promoter of several genes such as p16 (INK4a), RAR (retinoic acid receptor beta), human mutL homologue 1 (hMLH1) in cancer cell lines derived from colon, prostate and esophageal tumors (Fang et al., 2003). Hydralazine is an antihypertensive and vasodilator drug, recently known as a non-nucleoside DNMT inhibitor. This compound can induce cell death in p53-mutant leukemic T cells by a caspase-dependent mechanism (Ruiz-Magana et al., 2016). Hydralazine has been tested in different clinical cancer trials and in combination with valproate and chemotherapy showed clinical benefit in patients with refractory solid tumors (NCT00404508). Another non-nucleoside inhibitor is disulfiram, which can decrease genomic 5-methyl cytosine content, reactivating tumor suppressor genes silenced by hypermethylation, thus leading to a growth arrest of prostate cancer cells (Lin et al., 2011). Disulfiram has been tested in clinical trials for treatment of metastatic breast tumors (NCT03323346) and recurrent glioblastoma (NCT02678975). SGI-021 is a non-nucleoside DNMT inhibitor that exerts a potent anti-proliferative effect against cancer cell lines derived from breast cancer, histiocytic lymphoma, Burkitt’s lymphoma and prostate cancer (Datta et al., 2009).

### Histone deacetylases inhibitors

HDACs are considered targets for epigenetic cancer therapy and multiple diseases (Tang et al., 2013; West and Johnstone, 2014). FDA approved four categories of HDAC inhibitors: hydroxamic acids such as SAHA; cyclic peptides; benzamides and short-chain fatty acids (Nepali and Liou, 2021). SAHA, romidepsin, and tefinostat are HDAC inhibitors for class I HDAC and are in several clinical trials in breast (NCT 04190056); CTCL, PTCL (Barbarotta and Hurley, 2015), and HCC patients (NCT 02759601). Citarinostat (ACY-241), is a selective inhibitor of HDAC6, currently in phase Ib study for the treatment of NSLC patients (Awad et al., 2021). SIRT-1 is a HDAC classIII, known as sir-2-like proteins (sirtuin) has been identified in several tumors (breast, colorectal, prostate liver, pancreatic and lung cancer) and promotes cell migration, proliferation and chemoresistance of CSCs (Sun et al., 2013; Santolla et al., 2015; Pinho et al., 2016). A preclinical study demonstrated that SIRT-1-agonists (STACs) could represent a new therapeutic approach for the treatment of pancreatic cancer. Chini et al. (2016), in fact showed that use of STACs induced pancreatic cancer cells death through a process dependent by SIRT-1 and lysosomes. EX-527 (SEN0014196 or selisistat) is the most potent inhibitor of SIRT-1, tested in Huntigton’s disease patients in phase II clinical trial (Sussmuth et al., 2015). This inhibitor displays different biological effects on several tumor cells (Broussy et al., 2020). Broussy et al. showed that combination of two SIRT-1 inhibitors (EX-527 and nicotinamide) on lymphoma cells, induced apoptosis and cell cycle arrest (Broussy et al., 2020). Additional SIRT-1 inhibitors are nicotinamide, thioacyllysine, sirtinol, cambinol, splitomicin, suramin and tenovin (Hu et al., 2014). A clinical trial is ongoing to evaluate the effect of nicotinamide in patients with chronic lymphocytic leukemia (CLL) (NCT 04844528). Different clinical trials tested the effect of suramin in NSCLC (NCT01038752), hormone-refractory prostate cancer (NCT00002723) and adrenocortical cancer (NCT 00002921).

### Protein arginine methyltransferases inhibitors

Protein arginine methyltransferases (PRMTs) are enzymes that physiologically catalyze the methylation of R residues on histones. Dysfunction of PRMT is associated with the occurrence and progression of cancer (Mohammad et al., 2019). PRMT5 is a methyltransferase of class II that can catalyze symmetrical dimethylation and monomethylation, and is involved in signal transduction, cellular differentiation and RNA metabolism (Dacwag et al., 2007; Jansson et al., 2008; Bezzi et al., 2013). In cancer, PRMT5 is known to interact with cancer-associated deregulated pathways such as ErbB, FGF/FGFR signaling pathways and, for this reason, is considered a promising therapeutic target for the treatment of cancer (Sheng and Wang, 2016). PRMT5 is highly expressed in gastric cancer, breast cancer, glioblastoma and lymphoma. Different types of PRMT5 inhibitors have been identified. Bonday et al. (2018) showed that LLY-283, a PRMT5 inhibitor, exerted an anti-proliferative effect on several tumor cell lines. To date, a phase I study is ongoing to evaluate the effect of a selective PRMT5 inhibitor for the treatment of solid tumors and non-Hodgkin’s lymphoma (NCT 02783300).

### Demethylase inhibitors

Aberrant expression of different demethylases (LSDs or KDM1) and jumonji C (JmjC) groups of *N*-methyl-lysine demethylases (JmjC KDMs, KDM2–7) were identified in different tumors (McAllister et al., 2016). In particular, JmjC-KDMs depend on 2-oxoglutarate and oxygen availability (Tsai and Wu, 2014). It was shown that JmjC-KDMs are upregulated in hypoxia (Hancock et al., 2015), a common feature of solid tumors. Hoffmann et al. (2012) highlighted the presence of specific JmjC-KDMs, KDM3A, and KDM6B, in hypoxic tumors their expression has been correlated with cancer aggressiveness and development. To date, some compounds are currently been tested in clinical trials to inhibit LSD1 such as INCB059872 (NCT03514407) IMG-7289 (IMG) (NCT02273102), GSK2879552 (NCT02929498), CC90011 (NCT03850067), ORY-1001 (NCT02913443), while the development of JmjC-KDM specific inhibitors is more difficult due to their structural similarity and poor permeability (Morera et al., 2016).

## Concluding remarks

Aberrant epigenetic changes contribute to the transformation process of NSCs into CSCs. Specifically, epigenetic modifications sustain and maintain the self-renewal and stemness properties of NSCs, thus ultimately promoting the initiation and maintenance of CSCs by supporting their proliferation, invasiveness, metastatic potential and chemo-resistance capabilities.

Deepening the knowledge about the above-mentioned epigenetic mechanisms, thus identifying the most promising epigenetic druggable targets, would be useful for both the regenerative medicine and oncological settings.
